# Understanding the social dimensions of kidney care pathways: A scoping review protocol

**DOI:** 10.1371/journal.pone.0335597

**Published:** 2025-10-31

**Authors:** Reindolf Anokye, James Larkin, Abass Fehintola, Jessica Eustace-Cook, Rodrigo Martínez-Cadenas, Brett Duane, Bridget Johnston

**Affiliations:** 1 School of Nursing and Midwifery, Trinity College Dublin, the University of Dublin, Dublin, Ireland; 2 School of Dentistry, Trinity College Dublin, the University of Dublin, Dublin, Ireland; 3 Department of Nephrology and Hypertension, IIS-Fundación Jiménez Díaz UAM, Madrid, Spain; Public Library of Science, UNITED STATES OF AMERICA

## Abstract

**Objective:**

This scoping review aims to map the social dimensions of kidney care pathways.

**Introduction:**

End-stage kidney disease places significant demands on patients, caregivers, healthcare professionals, and the broader healthcare system. Kidney replacement therapy and conservative kidney management are the common management pathways for end stage kidney disease, but its delivery entails substantial time, financial, and social costs. Research has documented some social aspects of kidney care pathways, but findings are fragmented across disciplines and have not been synthesised coherently. A scoping review is therefore needed to map the existing evidence, identify key domains of social impact, and highlight gaps to inform future research and policy.

**Method:**

Eligible studies will include primary research that reports on the social dimensions of managing end-stage kidney disease (ESKD) in adults. Studies from all settings, involving relevant stakeholder groups including patients with end stage kidney disease, informal caregivers, healthcare staff, and Industrial and supply chain workers engaged in different roles associated with kidney replacement therapy and/or conservative kidney management, will be included. The review will follow JBI methodology. Peer-reviewed and grey literature will be identified through searches of electronic databases (MEDLINE, CINAHL, PsycInfo, SocINDEX, EconLit, Web of Science) and grey literature sources (including Google Scholar and organisational reports). Reference lists of relevant reviews and identified articles will also be screened to identify any other relevant articles. Two reviewers will independently screen records and extract data using a predefined form. Results will be synthesised narratively and presented in thematic and tabular formats.

**Discussion:**

This scoping review aims to generate evidence on the social impact of kidney replacement therapy and conservative management across multiple stakeholder groups. The review will provide a structured overview of how social outcomes are conceptualised, measured, and reported across the kidney care continuum.

**Review registration:**

Open Science Framework osf.io/63wpk.

## Introduction

Kidney disease is a significant public health issue [1]. Globally, more than 850 million people are estimated to have some form of kidney disease, more than twice the number affected by diabetes (422 million) and 20 times those with cancer (42 million) or HIV/AIDS (36.7 million), the majority unaware of their condition [2]. In 2017, kidney disease accounted for 1.2 million deaths worldwide, with its global prevalence increasing by 29.3% between 1990 and 2017 [3]. The burden of kidney disease is expected to rise due to population ageing and the growing prevalence of diabetes and hypertension [2,4].

End-stage kidney disease (ESKD) is associated with significant healthcare costs, morbidity, and mortality [5]. ESKD is characterised by permanent kidney failure requiring kidney replacement therapies, including dialysis and kidney transplantation or conservative kidney management [6,7]. The use of kidney replacement therapy is estimated to increase to 5.4 million by 2030 [8]. Conservative management focuses on symptom control, dietary modification [7], multidisciplinary care and palliative planning [9]. The treatment and management of ESKD can affect patients and those providing care and supportive therapies, with impacts extending to daily life, employment, and interpersonal relationships [10–12]. For example, dialysis requires regular treatment sessions, significant time commitment, and substantial personal and financial resources [13]. Kidney transplantation requires substantial preparation, including medical evaluation, donor matching, immunological testing, lifelong immunosuppressive therapy, frequent monitoring, and ongoing follow-up to ensure graft survival [14,15]. The production, distribution, and disposal of kidney care equipment may also contribute to labour exploitation, exacerbate inequities, and generate adverse environmental impacts [16,17].

Social impact assessment is the process of analysing, monitoring, and managing the intended and unintended, positive and negative social consequences of interventions, including any social change processes they initiate or influence among stakeholders [18,19]. The life-cycle perspective, formalised in Social Life Cycle Assessment (S-LCA), Social Life Cycle Assessment add-on (SOCA), and Product Social Impact Life Cycle Assessment (PSILCA), enables a systematic assessment of social and socio-economic impacts from raw material extraction through to end-of-life, supporting more equitable and sustainable decision-making [20–22].

A comprehensive understanding of the social impact of kidney care pathways is critical to advancing social responsibility and sustainability in the treatment and management of ESKD. To date, however, no review has systematically synthesised evidence on the social aspects of managing ESKD across diverse stakeholder groups (e.g., patients, caregivers, healthcare professionals, and supply chain workers). Existing reviews examining outcomes related to care pathways in ESKD have focused on specific treatment modalities (e.g., haemodialysis) or a limited set of social outcomes [23–25]. For example, a review by van der Mei et al. [25] examined only social participation and employment outcomes following kidney transplantation, whereas other reviews have focused more narrowly on the stressors experienced by dialysis staff [23,24]. This scoping review addresses existing gaps in the literature by incorporating (a) a wide range of patient and staff experiences, (b) the S-LCA framework, including information from the SOCA and PSILCA databases [20–22], and (c) additional approaches for assessing the social aspects of chronic conditions [25,26]. Using a comprehensive approach and established frameworks enables the identification of social hotspots within the kidney care pathway, thereby guiding interventions to mitigate negative impacts and enhance positive outcomes. The findings from this scoping review will contribute to building consensus on how to measure the social dimensions of care delivery and highlight opportunities to improve patient and staff experience as well as service delivery in kidney disease and other health conditions. Social factors also influence decision-making in healthcare [27]. As such, findings will inform policy recommendations and support healthcare planning for more sustainable and equitable kidney care provision. This review will map the available evidence, including how relevant outcomes are measured and reported, and the gaps in existing literature.

### Review question(s)

What are the reported experiences of adults with ESKD who are undergoing or have completed kidney replacement therapy or conservative kidney management?

What are the occupational, relational, and systemic stressors experienced by caregivers and healthcare workers related to kidney care delivery?

What are the reported social risks or impacts in the production, distribution, and disposal of equipment for kidney replacement therapy or conservative kidney management?

## Materials and methods

This scoping review will be conducted in accordance with the JBI methodology and reported following the Preferred Reporting Items for Systematic Reviews and Meta-Analyses extension for Scoping Reviews (PRISMA-ScR) [28,29].

### Inclusion

#### Population.

a) Adults with ESKD receiving kidney replacement therapy or conservative kidney management.
b) Informal caregivers and family members of adults with ESKD on kidney replacement therapy or conservative kidney management.
c) Healthcare and allied health professionals involved in ESKD care and/or supportive therapies, including nephrologists, nurses, dietitians, social workers, dialysis technicians, and transplant teams.
d) Industrial and supply chain workers engaged in raw material extraction, manufacturing, distribution, and waste management associated with kidney replacement therapy or conservative kidney management.

### Concept

This review examines the social impact of ESKD treatment through kidney replacement therapy or conservative kidney management, with a focus on outcomes that reflect the lived experiences of patients, caregivers, healthcare professionals, and industry stakeholders. The key domains include patient experience of care, social participation, and employment; caregiver psychosocial and economic burden; and occupational, relational, and systemic stressors across healthcare and supply chain contexts. **Table 1** outlines the core concepts, domains, and measurement time points.

**Table 1:** Domains, outcomes and measurement timepoints for concepts.

**Stage**	**Stakeholder**	**Social domains**	**Potential outcomes**
Pre-initiation (Period before starting kidney replacement therapy)/Assessment and shared decision-making	Patients	Social support, care environment, anticipated impact on employment and roles	• Patient satisfaction with care (i.e., whether care met expectations) • Patient experiences with staff (e.g., attentiveness of staff, pain management, clarity of discharge and medication communication, wait times, crowding, staff-patient interactions and relationships) • Physical care environment (i.e., extent to which care environment aligned with patient preferences) • Social support (e.g., providing material and financial assistance, guidance/information and emotional support [emotional expression, empathy]) • Challenges and opportunities for social interaction and paid work • Health and Safety (e.g., accidents, safety measures, disasters) • Child labour (e.g., children in employment) • Forced labour • Working time (e.g., hours of work) • Discrimination (e.g., gender wage gap, women in the labour force) • Social benefits, legal issues • Workers´ rights (e.g., trade union density, right of association) • Fair salary (e.g., living wage, minimum wage) • Financial (Financial strain due to medical costs, transport, or equipment, loss of income from reduced working hours or job loss, employment disruption, including career limitations or absenteeism) • Psychological and emotional, including psychological distress (e.g., anxiety, depression, emotional exhaustion), burnout and chronic caregiving stress, feelings of guilt, grief, or helplessness, worry about patient health, prognosis, or treatment complications, reduced emotional wellbeing and personal resilience. • Social and relational (Social isolation due to time constraints or emotional burden, strained relationships with partner, children, or extended family, role changes within the household or family unit, reduced engagement in leisure, social, or cultural activities) • Physical (fatigue and sleep disturbances, physical health decline due to stress-related illness, and neglect of personal health needs).
	Healthcare Staff	Care planning, team coordination, organisational support, gender roles; labour conditions	
	Informal Caregivers/ Family	Anticipatory support roles, preparation for care, social coordination	
	Industrial/Supply Chain Workers	Production and logistics planning, coordination with healthcare; gender roles; labour conditions	
Initiation (Early phase of KRT where patients adapt to treatment routines; staff coordinate care delivery, and industrial/supply chain systems ensure availability of resources.	Patients	Social participation/employment, care environment orientation	
	Healthcare Staff	Care delivery, team communication, workload, gender roles; labour conditions	
	Informal Caregivers/ Family	Engagement in care delivery, coordination with healthcare staff	
	Industrial/Supply Chain Workers	Resource availability, logistical support, workplace equity and safety, gender roles; labour conditions	
Maintenance (Ongoing treatment phase)/ Active conservative management	Patients	Sustaining social roles/employment, continuity of care, support networks	
	Healthcare Staff	Routine care, team coordination, occupational support, workforce rights and inclusion, gender roles; labour conditions	
	Informal Caregivers/ Family	Ongoing care provision, social support, coordination	
	Industrial/Supply Chain Workers	Routine production/distribution, occupational safety; workforce rights and inclusion, regulatory compliance; social and ethical responsibilities, gender roles; labour conditions	
Complications (Period marked by illness-related or treatment-related challenges)	Patients	Increased social support needs, care environment adaptation, employment	
	Healthcare Staff	Complex care coordination, occupational stress, gender roles, labour conditions	
	Informal Caregivers/ Family	Intensified caregiving, social and financial burden	
	Industrial/Supply Chain Workers	Response to disruptions, workload, workplace pressures and rights, safe disposal and regulatory compliance; social and ethical responsibilities, gender roles; labour conditions	
Palliative/ Withdrawal (Final phase when KRT is withdrawn or palliative care is provided)	Patients	Social support for end-of-life, care environment focus	
	Healthcare Staff	Palliative care provision, coordination, gender roles; labour conditions	
	Informal Caregivers/ Family	End-of-life care, emotional and social support	
	Industrial/Supply Chain Workers	Safe disposal and regulatory compliance; social and ethical responsibilities, gender roles; labour conditions	

KRT: Kidney Replacement Therapy.

### Context

This review focuses on kidney care pathways related to kidney replacement therapy or conservative kidney management, encompassing individuals receiving treatment, those providing informal or formal care, and those supporting kidney care systems.

a) Receiving kidney care: Adults with ESKD who are eligible for, undergoing, or have completed kidney replacement therapy or conservative kidney management, across all care settings (e.g., in-centre, home-based, public, or private facilities).
b) Providing informal or formal care: Family caregivers and healthcare professionals (e.g., nephrologists, nurses, and allied health staff) involved in the delivery of kidney replacement therapy or conservative kidney management and care of adults with ESKD.
c) Supporting kidney care systems: Workers engaged in the production, distribution, or disposal of equipment and materials associated with kidney replacement therapy or conservative kidney management.

All stages of care will be considered, including outcomes assessed at initiation, maintenance, and follow-up, across different care settings (home-based and facility-based; public and private institutions), and at any time point (pre-treatment, during treatment, or post-treatment). Evidence will be drawn from diverse disciplines, including clinical and public health sciences, social sciences, environmental sciences, biosciences, engineering, policy, law, and education.

### Exclusion

Studies focusing on children or adolescents (<18 years), kidney donors, or animals.

Studies primarily evaluating the effects of interventions unrelated to kidney replacement therapy (e.g., cognitive behavioural therapy, exercise interventions).

### Types of sources

Peer-reviewed journal articles (e.g., quantitative studies, qualitative studies, mixed-methods studies) and Clinical Guidelines and Policy Reports (e.g., documents from organisations like the Kidney Disease Improving Global Outcomes (KDIGO), World Health Organisation (WHO), and national health agencies) will be included. Other non-peer-reviewed articles (e.g., government and NGO reports, white papers, and unpublished research from kidney foundations, patient advocacy groups, and healthcare workforce studies) will be considered.

Commentaries, editorials, discussion papers and study protocols will be excluded. Any other studies not using primary data or not reporting outcomes based on primary data (e.g., systematic or scoping reviews) will also be excluded.

### Search strategy

A three-step strategy will be followed [30]. An initial exploratory search of MEDLINE via EBSCOhost (S1 Table) will be undertaken to identify key articles on the topic. The next step will involve generating a list of keywords from the words in the titles, abstracts, and indexed terms of the identified articles, which will be used to develop a comprehensive search strategy. Keywords will be used to search across all selected sources and databases.

Grey literature sources will be searched to identify relevant literature. We will keep our search strategy reproducible and use search terms consistently between different sources. To limit the screening process, there will be a set number of pages (e.g., first 100 results). The name of the sources, URL, the date searched, and the search terms used will be reported.

References of the selected papers for full-text review and those included in the scoping review will be screened for additional relevant studies. Forward citation tracking in Google Scholar, Scopus, and Web of Science will be conducted to identify more recent studies citing key articles. Authors of included studies will be contacted for further information if deemed relevant.

There will be no restrictions on language or publication status (i.e., published, unpublished, in press, in progress, preprint) or publication date. Documents published in languages other than English that meet the inclusion criteria will be reviewed by a fluent speaker and/or translated.

We will search MEDLINE, CINAHL, PsycInfo, SocINDEX with Full Text, EconLit, Web of Science, and Business Source Complete to identify relevant studies. Non-peer-reviewed studies/grey literature sources, including Open Grey and Google Scholar (first 100 pages), will be searched. The search strategies will be published with the review as supplementary materials.

### Study selection

Citations and search results will be collated and imported into EndNote for duplicate removal. The deduplicated records will then be transferred to Covidence for screening. Two reviewers will independently screen all titles, abstracts, and full texts for inclusion, and any disagreements will be resolved by a third reviewer.

### Data extraction

Two authors will independently extract relevant data using data extraction form (S2 Table). Data will be entered in Microsoft Excel spreadsheet. The results will be organised according to the review questions and thematic categories determined by the research team. An iterative process will be employed, allowing for continuous updates as new data emerges. Extracted data will include study characteristics (e.g., author details, year, study design, data collection instruments, and study location) and outcomes related to the social impact of kidney care pathways.

### Presentation of data and analysis

Data will be synthesised using a narrative approach, consistent with the JBI methodology for scoping reviews [28,29]. Quantitative and qualitative data will be analysed separately and presented side by side to reflect the scope and nature of the evidence on the social impact of dialysis and kidney transplantation across stakeholder groups.

Quantitative data (e.g., frequencies, proportions, and descriptive statistics) will be summarised using tables and figures to characterise study designs, populations, interventions, outcomes, and reported social impact domains.

Qualitative data (e.g., themes from interviews, focus groups, or open-ended survey responses) will be analysed using basic thematic analysis. Themes will be developed inductively and mapped to predefined conceptual domains related to patient experiences, caregiver burden, occupational stress, and supply chain impacts.

Findings from both data types will be displayed in structured tables and thematic maps, allowing for comparison across stakeholder groups, care settings, and phases of the kidney care pathway. No formal synthesis or integration will be conducted (S3 Table). This approach supports the goal of scoping the breadth and diversity of evidence on social outcomes, without aggregating findings or assessing effect sizes [29].

### Study status

Start date of search: August 2025; anticipated date of completing review: February 2026

#### Current study status.

Preliminary searches: Yes

Piloting search strategy: Yes

Pilot screening of search results: Yes

Formal screening of search results against eligibility criteria: Started

Data extraction: Not started

Data analysis and interpretation: Not started

## Discussions and conclusion

This scoping review aims to map the extent, range, and nature of evidence on the social impact of kidney replacement therapy and conservative management across multiple stakeholder groups. By systematically identifying and synthesising outcomes related to patient experiences, caregiver burden, workforce stressors, and the ethical dimensions of kidney care supply chains, the review will address an important gap in understanding the broader consequences of end-stage kidney disease management. The inclusion of both peer-reviewed and grey literature, and frameworks such as S-LCA and data from SOCA and PSILCA databases, will allow for a more comprehensive and interdisciplinary appraisal of social outcomes across care pathways.

However, several limitations should be acknowledged. First, as a scoping review, this study will not evaluate the quality or risk of bias of included studies and intervention effectiveness or causal relationships, limiting their utility for informing evidence-based interventions without further synthesis. Second, literature on supply chain and industrial workers in kidney care may be sparse or poorly reported, leading to gaps in stakeholder coverage and a patient- and provider-centric evidence base. Furthermore, grey literature often lacks rigorous peer review, comprehensive reporting, or methodological transparency, affecting the reliability of some sources. Also, given the breadth of topics and outcomes covered, ensuring consistent data extraction across reviewers may be challenging, particularly where studies report social outcomes indirectly or anecdotally.

Despite these limitations, the review will provide a structured overview of how social outcomes are conceptualised, measured, and reported across the kidney care continuum. We will highlight methodological and conceptual gaps, such as underreporting of supply chain impacts or insufficient attention to areas such as exploitation, discrimination and inequities (e.g., gender, socioeconomic status, migrant status), which may guide future research agendas. By reporting the lived experiences and systemic impacts often overlooked in traditional outcome frameworks and existing evidence synthesis, findings from the scoping review will support efforts to design more equitable, responsive, and sustainable models of care.

## Supporting information

S1 Table Search strategy (MEDLINE via EBSCOhost). (DOCX)

S2 Table Draft data extraction form. (DOCX)

S3 Table PRISMA-P checklist from Moher et al., (2016). (DOCX)
