# Supplementary material for: Understanding the social dimensions of kidney care pathways: A scoping review protocol
Source: PLoS One. 2025 Oct 31;20(10):e0335597. doi: 10.1371/journal.pone.0335597 (PMC12578194; doi:10.1371/journal.pone.0335597)
Supplement: S1 Table — (DOCX) [file pone.0335597.s001.docx]

**Supplementary Table 1: Search Strategy (MEDLINE via EBSCOhost)**

|  | Fri, August 22, 2025 9:37:03 a.m. | |  |  |  |  |
| --- | --- | --- | --- | --- | --- | --- |
| **#** | | | **Query** | **Limiters/Expanders** | **Last Run Via** | **Results** |
| S10 | | | S4 AND S6 AND S9 | Expanders - Apply equivalent subjects  Search modes - Proximity | Interface - EBSCOhost Research Databases  Search Screen - Advanced Search  Database - MEDLINE | 3,372 |
| S9 | | | S7 OR S8 | Expanders - Apply equivalent subjects  Search modes - Proximity | Interface - EBSCOhost Research Databases  Search Screen - Advanced Search  Database - MEDLINE | 3,037,794 |
| S8 | | | XB (nurse* OR caregiver* OR carer* OR nephrologist* OR famil* OR spous* OR physician* OR “medical supplier*” OR (health* OR social* OR medical* OR clinical* OR industry* OR manufactur* OR production* OR supply* OR waste*) N2 (staff* OR worker* OR professional* OR technician*)) | Expanders - Apply equivalent subjects  Search modes - Proximity | Interface - EBSCOhost Research Databases  Search Screen - Advanced Search  Database - MEDLINE | 2,462,144 |
| S7 | | | (MH "Nurses+") OR (MH "Allied Health Personnel") OR (MH "Health Personnel+") OR (MH "Nursing Staff+") OR (MH "Physicians+") OR (MH "Nursing Staff, Hospital+") OR (MH "Community Health Services+") OR (MH "Nephrologists") OR (MH "Nephrology Nursing") | Expanders - Apply equivalent subjects  Search modes - Proximity | Interface - EBSCOhost Research Databases  Search Screen - Advanced Search  Database - MEDLINE | 968,249 |
| S6 | | | S3 OR S5 | Expanders - Apply equivalent subjects  Search modes - Proximity | Interface - EBSCOhost Research Databases  Search Screen - Advanced Search  Database - MEDLINE | 2,032,207 |
| S5 | | | XB (((social* OR “mental stress*”) N2 (impact* OR outcome* OR implication*)) OR ((work* OR job* OR employment* OR career* OR occupation*) N2 (stress* OR burnout)) OR turnover* OR exploit* OR abus* OR “human rights” OR “return to work*”) | Expanders - Apply equivalent subjects  Search modes - Proximity | Interface - EBSCOhost Research Databases  Search Screen - Advanced Search  Database - MEDLINE | 552,910 |
| S4 | | | S1 OR S2 | Expanders - Apply equivalent subjects  Search modes - Proximity | Interface - EBSCOhost Research Databases  Search Screen - Advanced Search  Database - MEDLINE | 487,567 |
| S3 | | | (MH "Stress, Psychological+") OR (MH "Conflict, Psychological+") OR (MH "Compassion Fatigue") OR (MH "Burnout, Psychological+") OR (MH "Burnout, Professional") OR (MH "Occupational Stress") OR (MH "Return to Work") OR (MH "Social Support+") OR (MH "Treatment Outcome") | Expanders - Apply equivalent subjects  Search modes - Proximity | Interface - EBSCOhost Research Databases  Search Screen - Advanced Search  Database - MEDLINE | 1,518,357 |
| S2 | | | XB ((chronic* OR transplant* OR disease* OR replac*) N2 (kidney* OR renal*) OR dialys* OR hemodialys* OR haemodialys* OR “kidney care” OR hemofiltrat* OR haemofiltrat*) | Expanders - Apply equivalent subjects  Search modes - Proximity | Interface - EBSCOhost Research Databases  Search Screen - Advanced Search  Database - MEDLINE | 470,928 |
| S1 | | | (MH "Dialysis+") OR (MH "Kidney Transplantation) OR (MH "Kidney Failure, Chronic+") OR (MH "Renal Insufficiency, Chronic+") | Expanders - Apply equivalent subjects  Search modes - Proximity | Interface - EBSCOhost Research Databases  Search Screen - Advanced Search  Database - MEDLINE | 24,353 |
