# Supplementary material for: Understanding the social dimensions of kidney care pathways: A scoping review protocol
Source: PLoS One. 2025 Oct 31;20(10):e0335597. doi: 10.1371/journal.pone.0335597 (PMC12578194; doi:10.1371/journal.pone.0335597)
Supplement: S2 Table — (DOCX) [file pone.0335597.s002.docx]

**Supplementary Table 2: Draft data extraction form**

| **Category** | **Data Item** |
| --- | --- |
| Bibliographic Information | Author(s) |
|  | Year |
|  | Title |
|  | Country/Region |
| Study Characteristics | Study design/type (quantitative, qualitative, mixed methods) |
|  | Setting (e.g., hospital, community) |
| Population | Stakeholder group (e.g., patient, caregiver, healthcare worker, industrial/supply chain worker) |
|  | Sample size |
|  | Age range |
|  | Gender distribution (% female) |
| Kidney Care Context | Type of intervention (dialysis, transplantation, or both) |
|  | Stage of care (e.g., initiation, maintenance, post-transplant) |
|  | Care setting (e.g., home, centre-based, public, private) |
| Data Collection | Instruments or methods used (e.g., interviews, surveys, administrative data) |
| Reported Social Outcomes (X if applicable) | Patient experiences (Occupational, relational, systemic stressors, healthcare related) |
|  | Staff experience (Occupational, relational, and systemic stressors) |
|  | Caregiver experiences (Occupational, relational, and systemic stressors) |
| Outcome Details | Specific outcomes reported (e.g., satisfaction, burnout, social support) |
|  | Quotes or illustrative findings (qualitative) |
|  | Quantitative data (frequencies, proportions) |
| Key Findings | Summary of main results and conclusions |
| Notes / Comments | Methodological limitations or contextual notes |
